# Supplementary material for: Phylogenetic analysis of 17271 Indian SARS-CoV-2 genomes to identify temporal and spatial hotspot mutations
Source: PLoS One. 2022 Mar 28;17(3):e0265579. doi: 10.1371/journal.pone.0265579 (PMC8959188; doi:10.1371/journal.pone.0265579)
Supplement: S1 File — (PDF) [file pone.0265579.s001.pdf]

Supplementary to "Phylogenetic Analysis of 17271 Indian SARS-CoV-2 Genomes to Identify Temporal and Spatial Hotspot Mutations"

Nimisha Ghosh[a,c], Suman Nandi[b,c], Indrajit Saha[b,c,*]

[a]*Department of Computer Science and Information Technology, Institute of Technical Education and Research, Siksha 'O' Anusandhan (Deemed to be University), Bhubaneswar, Orissa, India*
[b]*Department of Computer Science and Engineering, National Institute of Technical Teachers' Training and Research, Kolkata, West Bengal, India*
[c]*Equally contributed*

*Corresponding author: indrajit@nitttrkol.ac.in

Table S1: List of Temporal Hotspot Mutations for Indian SARS-CoV-2 Genomes

Genomic	Change in	Change in	Gene	January-March 2020	April 2020	May 2020	June 2020	July 2020	August 2020	September 2020	October 2020	November 2020	December 2020	January 2021	February 2021	March 2021	April 2021	May 2021	June 2021	July 2021	August 2021	September 2021
Position	Nucleotide	Amino Acid		191	441	977	1062	683	632	629	380	452	983	500	980	1907	3054	2408	1293	632	15	52
313	C>T	Synonymous	NSP1	0.12021	0.43636	0.54277	0.54631	0.66456	0.6079	0.59785	0.27517	0.27704	0.21822	0.14119	0.28234	0.164	0.02363	0.01228	0.01154	0	0	0
1397	G>A	V198I	NSP2	0.53043	0.05149	0.03733	0.00749	0.04318	0.01175	0	0	0	0.00801	0	0	0	0	0	0.00631	0	0	0
1947	T>C	V381A	NSP2	0.13318	0.11629	0.24131	0.41328	0.42678	0.46034	0.40164	0.49862	0.66982	0.71371	0.42311	0.29536	0.11592	0.02567	0	0	0	0	0
3037	C>T	Synonymous	NSP3	0.63531	0.70595	0.45544	0.15842	0.14696	0.11196	0.08787	0.12119	0.04396	0.02069	0.02599	0.02074	0.02673	0.00841	0.03137	0.01154	0.02132	0.36221	0.15841
3267	C>T	T183I	NSP3	0	0	0.00806	0.04879	0.23846	0.35065	0.25311	0.36776	0.48539	0.49084	0.63605	0.63356	0.64996	0.35788	0.04562	0.02952	0	0	0
4181	G>T	A488S	NSP3	0	0	0	0.00749	0.01099	0	0	0	0	0.01462	0.02875	0.03724	0.05091	0.33963	0.63932	0.68881	0.68595	0.69142	0.57228
5184	C>T	P822L	NSP3	0.03244	0	0.01469	0.00749	0	0	0	0.04589	0.06061	0.05024	0.01438	0.07375	0.34355	0.69964	0.66981	0.68717	0.68595	0.67749	0.60769
5700	C>A	A994D	NSP3	0.03244	0.37951	0.50567	0.53036	0.65981	0.60235	0.59193	0.26659	0.28161	0.19173	0.12017	0.27737	0.1718	0.02817	0.01228	0.01154	0	0	0.09222
6312	C>A	T1198K	NSP3	0.59276	0.6678	0.44906	0.08942	0.12725	0.02132	0.0118	0	0.02829	0.04718	0.06479	0.00803	0.00824	0.01991	0.06152	0.1135	0.02132	0	0
6402	C>T	P1228L	NSP3	0	0	0.01469	0.02474	0.01996	0.03825	0.0214	0.01818	0	0.03188	0.01438	0.03196	0.04842	0.34419	0.64548	0.67954	0.68595	0.69142	0.57228
7124	C>T	P1469S	NSP3	0	0	0	0	0	0	0	0	0.01568	0.02263	0.01438	0.03724	0.06737	0.34103	0.6399	0.68529	0.68595	0.69142	0.57228
8917	C>T	Synonymous	NSP4	0.05766	0.14791	0.16742	0.29869	0.21017	0.27599	0.3893	0.57761	0.3559	0.29388	0.5075	0.27737	0.08939	0.01	0.00365	0.02094	0.01175	0	0
8986	C>T	Synonymous	NSP4	0	0	0	0	0	0	0	0.01818	0	0	0.01438	0.03196	0.05336	0.34439	0.63584	0.68468	0.68595	0.69142	0.57228
9053	G>T	V167L	NSP4	0	0	0	0	0	0	0	0	0	0	0.01438	0.03724	0.05091	0.34517	0.64077	0.69268	0.68595	0.69142	0.57228
9389	G>A	D279N	NSP4	0	0.07172	0.10751	0.17222	0.13194	0.19991	0.35501	0.55503	0.33276	0.27678	0.4937	0.26982	0.08939	0	0	0	0	0	0
9891	C>T	A446V	NSP4	0	0	0	0	0.01099	0	0.0118	0.01818	0.03975	0.0567	0.05092	0.06145	0.30477	0.69886	0.69625	0.69617	0.68066	0.67749	0.52971
10029	C>T	T492I	NSP4	0	0	0	0.02474	0.03586	0.11196	0.08141	0.05816	0.02829	0.07023	0.07343	0.06145	0.06266	0.34228	0.64251	0.68529	0.68595	0.69142	0.57228
11083	G>T	L37F	NSP6	0.82391	0.79874	0.44044	0.1477	0.22996	0.23915	0.3212	0.26868	0.30325	0.35872	0.18601	0.25104	0.08516	0.11195	0.12619	0.1245	0.14081	0.22372	0
11201	A>G	T77A	NSP6	0	0	0	0.00749	0	0	0.0384	0.03272	0.03975	0.04718	0.08177	0.57057	0.67151	0.64406	0.66818	0.69311	0.68556	0.69142	0.57228
11296	T>G, T>-	F108L/-	NSP6	0	0	0	0.00749	0	0	0	0	0.03975	0.18104	0.21	0.49698	0.82674	0.41274	0.05058	0.02531	0	0	0
11332	A>G	Synonymous	NSP6	0	0	0	0.00749	0	0	0	0	0	0	0.01438	0.02648	0.04842	0.3414	0.65751	0.67954	0.68595	0.69142	0.57228
11418	T>C	V149A	NSP6	0	0	0	0	0	0	0	0.03272	0.06061	0.03188	0.03656	0.12592	0.29544	0.70573	0.70413	0.70538	0.68066	0.64925	0.52971
13730	C>T	A97V	RdRp	0.58269	0.66587	0.48254	0.11065	0.11624	0.04605	0.03017	0.04589	0.02829	0	0.02599	0	0.00824	0.00295	0.01228	0.01154	0	0	0
14408	C>T	P323L	RdRp	0.63531	0.6971	0.54115	0.24011	0.19513	0.06073	0.07478	0.08082	0.12996	0.07457	0.07343	0.04234	0.05818	0.03042	0.01489	0.02952	0.06073	0.6058	0.15841
18877	C>T	Synonymous	Exon	0.21862	0.38814	0.5533	0.66299	0.66359	0.6079	0.56622	0.6463	0.68886	0.67905	0.70613	0.61121	0.24152	0.04564	0.00957	0.02952	0.02132	0	0
19220	C>T	A394V	Exon	0	0	0	0.00749	0	0	0.0118	0	0	0	0.01438	0.03196	0.04591	0.34019	0.64257	0.67923	0.68595	0.69142	0.57228
21034	C>T	Synonymous	NSP16	0	0	0.00806	0.06361	0.25822	0.42345	0.28156	0.42456	0.45989	0.46247	0.61845	0.47337	0.1565	0.02113	0.01593	0.02952	0.03005	0	0
21846	C>T	T95I	Spike	0	0	0	0	0.01996	0.02132	0.03017	0.03272	0.08595	0.07457	0.08986	0.41862	0.55193	0.51835	0.54572	0.58027	0.67811	0.54559	0.69315
21987	G>A, G>-	G142D/-	Spike	0	0	0	0.01937	0	0	0	0	0.02829	0.04789	0.05473	0.62506	0.80846	0.87431	0.76579	1.0067	0.93091	0.54559	0.3085
22022	G>A	E154K	Spike	0	0	0	0	0	0	0.03017	0.03272	0.03975	0.06178	0.0608	0.55027	0.76572	0.54036	0.16868	0.09666	0.01175	0	0
22030	G>-	E156-	Spike	0	0.01601	0	0	0	0	0	0	0	0.01462	0.01438	0.12227	0.29242	0.69303	0.68573	0.51234	0.45393	0.36221	0.21456
22032	T>-	F157-	Spike	0	0	0	0	0	0	0	0.01818	0	0.02069	0.01438	0.18791	0.3078	0.756	0.49521	0.39171	0.45393	0.36221	0.21456
22033	C>-	F157-	Spike	0	0	0	0	0	0.01175	0	0.03272	0.05962	0.03714	0.04036	0.13028	0.30656	0.76607	0.48689	0.48015	0.45393	0.36221	0.21456
22034	A>G, A>-	R158G/-	Spike	0	0	0	0	0	0	0	0.03635	0.02829	0.03989	0.03656	0.12592	0.3184	0.89681	0.81429	0.81496	0.49851	0.36221	0.21456
22444	C>T	Synonymous	Spike	0.18848	0.27076	0.46277	0.67332	0.60236	0.62652	0.57955	0.64744	0.75515	0.71598	0.69315	0.6458	0.31331	0.12975	0.03917	0.01154	0.03005	0	0
23012	G>C, G>A	E484Q/K	Spike	0.03244	0	0.00806	0.02116	0.01996	0	0.07676	0.08356	0.06802	0.08909	0.20153	0.82687	0.88114	0.5958	0.18006	0.10388	0.05779	0.22372	0.34883
23403	A>G	D614G	Spike	0.63531	0.69774	0.48027	0.15097	0.15184	0.03825	0.05373	0.04589	0.02829	0.01462	0.02599	0.02074	0.01172	0.0109	0.00672	0.01154	0.02132	0.36221	0.15841
23604	C>A, C>G	P681H/R	Spike	0	0	0	0.00749	0.0612	0.04605	0.25954	0.21551	0.24915	0.43913	0.54015	1.02071	1.01066	0.4497	0.07978	0.05045	0.02132	0.36221	0.15841
23929	C>T	Synonymous	Spike	0.63088	0.65279	0.39016	0.10448	0.1107	0.05352	0.03017	0	0.03975	0	0.01438	0.00803	0.00824	0.01	0.01593	0	0	0	0
24410	G>A	D950N	Spike	0	0	0	0.00749	0	0	0	0	0	0	0.01438	0.14322	0.39406	0.8167	0.66129	0.37805	0.5724	0.67749	0.68696
24775	A>T, A>-	Q1071H/-	Spike	0	0	0	0	0	0	0	0	0	0.02263	0.06252	0.69608	0.80534	0.58293	0.12367	0.0589	0.01175	0	0
25469	C>T	S26L	ORF3a	0	0	0	0	0	0	0.0384	0.05816	0.07963	0.06581	0.08177	0.65125	0.77293	0.43958	0.07952	0.04525	0.03306	0.36221	0.15841
25563	G>T	Q57H	ORF3a	0.24675	0.3966	0.64183	0.67187	0.69252	0.62045	0.60326	0.64465	0.68439	0.68512	0.70613	0.62884	0.25548	0.05958	0.03132	0.01784	0.01175	0	0
26060	C>T	T223I	ORF3a	0	0.06183	0.11887	0.06596	0.09403	0.14007	0.25634	0.2554	0.45174	0.56206	0.43528	0.27236	0.13505	0.02385	0.01228	0	0.02132	0	0
26735	C>T	Synonymous	Membrane	0.18848	0.38385	0.56685	0.6606	0.65467	0.61193	0.56103	0.6463	0.68657	0.67871	0.69315	0.60948	0.24063	0.04589	0.0246	0.02094	0.01175	0	0.09222
26767	T>C, T>G	I82T/S	Membrane	0	0	0	0	0	0	0.0214	0.05089	0.07872	0.05827	0.06252	0.4939	0.84724	0.89284	0.29328	0.27597	0.06309	0.36221	0.15841
27874	C>T	T40I	ORF7b	0	0.02889	0.00806	0	0.01099	0.02132	0	0.01818	0.01568	0.00801	0.03656	0.03724	0.06503	0.34509	0.64491	0.70016	0.68556	0.69142	0.59084
28248	G>-	D119-	ORF8	0	0	0.02655	0.00749	0	0	0	0.01818	0	0.02069	0.01438	0.03539	0.22758	0.71357	0.6589	0.60988	0.60637	0.36221	0.21456
28249	A>T, A>-	D119V/-	ORF8	0	0	0.0346	0.00749	0	0	0	0.01818	0.01568	0.0287	0.01438	0.04721	0.2261	0.84388	0.81342	0.81706	0.7564	0.69142	0.21456
28250	T>-	D119-	ORF8	0	0.01601	0.02655	0.00749	0.01099	0.01175	0	0	0	0.01462	0.02875	0.04661	0.2115	0.69031	0.66012	0.65243	0.711	0.57833	0.3085
28251	T>-	F120-	ORF8	0	0.01601	0.0208	0.01498	0.02815	0.01175	0.04623	0.03272	0.01568	0.06169	0.04644	0.05524	0.22263	0.69728	0.6755	0.65749	0.71349	0.36221	0.3085
28252	T>-	F120-	ORF8	0	0.01601	0.0208	0.00749	0.01996	0.01175	0	0	0	0.02069	0.03656	0.03451	0.21	0.68974	0.66666	0.65243	0.70261	0.36221	0.3085
28253	C>A, C>T, C>-	F120L/F/-	ORF8	0	0.09067	0.04733	0.02116	0.04684	0.08425	0.12958	0.14863	0.17719	0.26801	0.23048	0.27896	0.38521	1.13895	1.08851	0.98317	0.87833	0.70869	0.37081
28280	G>T, G>C	D3Y/H	Nucleocapsid	0	0	0	0	0.01099	0.01175	0.06097	0.09145	0.15029	0.24273	0.27059	0.66855	0.91893	0.53968	0.10384	0.0585	0	0	0
28311	C>T	P13L	Nucleocapsid	0.64212	0.71328	0.39439	0.09332	0.1107	0.03005	0.12414	0.06976	0.01568	0.00801	0	0.09549	0.00824	0.00778	0.00957	0.01261	0	0	0
28688	T>C	Synonymous	Nucleocapsid	0.57987	0.06183	0.03733	0.00749	0.04318	0	0	0	0	0.02641	0	0	0	0	0	0	0	0	0
28854	C>T	S194L	Nucleocapsid	0.22066	0.28729	0.46105	0.6393	0.61568	0.61586	0.56792	0.64124	0.69762	0.67728	0.69286	0.61035	0.25039	0.04323	0.01854	0.02724	0	0	0
28881	G>A, G>T	R203K/M	Nucleocapsid	0.38864	0.53127	0.66198	0.72623	0.86601	0.79095	0.7396	0.78752	0.74527	0.71656	0.82738	1.13342	1.03262	0.47406	0.15294	0.06606	0.03306	0.36221	0.15841
28882	G>A	Synonymous	Nucleocapsid	0.38864	0.53127	0.66198	0.69816	0.85618	0.78061	0.68911	0.70769	0.66551	0.65134	0.71685	0.68897	0.74392	0.3821	0.06096	0.03354	0.01175	0	0
28883	G>C	G204R	Nucleocapsid	0.38864	0.53127	0.66198	0.71049	0.85615	0.78919	0.67924	0.70769	0.66551	0.67009	0.70225	0.67724	0.74249	0.37987	0.05425	0.02094	0.01175	0	0
28916	G>T	G215C	Nucleocapsid	0.03244	0.02889	0	0	0	0	0.0214	0	0.01568	0.01462	0.04036	0.06306	0.05091	0.33929	0.65133	0.69472	0.68556	0.69142	0.55193
29402	G>T	D377Y	Nucleocapsid	0	0.01601	0.04265	0.02686	0.01996	0.03825	0.10643	0.11159	0.21317	0.18421	0.20573	0.64017	0.70951	0.40367	0.09233	0.05678	0.05955	0.36221	0.15841

Table S2: List of Spatial Hotspot Mutations for Indian SARS-CoV-2 Genomes

Genomic Position	Change in Nucleotide	Change in Amino Acid	Gene	Maharashtra	Telangana	Gujarat	West Bengal	Delhi	Andhra Pradesh	Karnataka	Rajasthan	TamilNadu	Punjab	Chhattisgarh	Manipur	Odisha	Uttar Pradesh	Haryana	Himachal Pradesh	Sikkim	Jammu and Kashmir	Puducherry	Meghalaya	Uttarakhand	Madya Pradesh	Kerala	Chandigarh	Assam
				3674	2506	2333	1637	1240	1077	520	434	423	418	364	270	238	229	193	184	165	164	138	135	126	109	106	102	101
884	C>T	R27C	NSP2	0.01674	0	0.0153	0.00941	0	0.0074	0.06276	0	0.01659	0	0	0	0	0	0	0	0	0	0	0	0	0	0.6155	0	0
1191	C>T	P129L	NSP2	0.26138	0.41622	0.3376	0.60894	0.39589	0.59632	0.24594	0.172	0.57428	0.18425	0.65426	0.49836	0.37821	0.44465	0.23416	0.13718	0.63789	0.36649	0.62794	0.61024	0.27417	0.18366	0.26405	0	0.58325
1397	G>A	V198I	NSP2	0.01211	0.00704	0.01789	0.00513	0	0	0.06276	0	0	0.01676	0	0	0	0	0	0	0	0	0	0	0	0	0.6299	0	0
1947	T>C, T>-	V381A/-	NSP2	0.08909	0.0192	0.20756	0.14894	0.68719	0.01914	0.06276	0.59878	0.01659	0.30364	0.27014	0.16934	0.14614	0.25624	0.63651	1.00628	0.47085	0.62632	0.04241	0	0.77067	0.2814	0.21456	0.62482	0.43186
3037	C>T	Synonymous	NSP3	0.08778	0.16499	0.08842	0.14069	0.24941	0.09594	0.21456	0.16588	0.26118	0.13804	0.06023	0.04345	0.29672	0.2793	0.30784	0.05943	0.0649	0.21075	0.07488	0.0762	0.29304	0.30969	0.69298	0.19281	0.22213
5184	C>T	P822L	NSP3	0.50335	0.58673	0.60365	0.64737	0.43562	0.66757	0.34213	0.28416	0.63867	0.23218	0.64728	0.68705	0.4369	0.71401	0.31941	0.1936	0.69098	0.33035	0.68486	0.6736	0.36128	0.21028	0.28684	0.05423	0.668
6402	C>T	P1228L	NSP3	0.30596	0.45503	0.43639	0.11019	0.10192	0.58479	0.51994	0.142	0.58616	0.0538	0.08365	0.68705	0.09414	0.69118	0.23118	0.03346	0.40081	0.13516	0.56234	0.47514	0.04569	0	0.05257	0	0.29643
8653	G>T	M33I	NSP4	0.00463	0.01791	0.0153	0.01337	0	0.0074	0.06276	0	0	0.01676	0.06023	0	0	0.05574	0	0	0	0	0	0.0762	0	0	0.6299	0	0
8917	C>T	Synonymous	NSP4	0.0366	0.09779	0.06871	0.29755	0.14506	0.15244	0.60824	0.50918	0.44798	0.0538	0.15343	0.13269	0.57992	0.04974	0	0.16035	0	0.0368	0.15408	0	0	0	0.36766	0.13071	0.33976
9053	G>T	V167L	NSP4	0.30343	0.45248	0.42696	0.11019	0.09228	0.5857	0.51994	0.1341	0.58616	0.0538	0.1154	0.68531	0.09414	0.74627	0.23118	0	0.40081	0.06521	0.56234	0.46401	0.04569	0	0.05257	0	0.29643
9389	G>A	D279N	NSP4	0.03379	0.09467	0.04725	0.29181	0.12261	0.12371	0.44746	0.50294	0.29568	0.04241	0.15343	0.13269	0.55771	0	0	0.16035	0	0.0368	0.15408	0	0	0	0.24002	0.09503	0.29643
9891	C>T	A446V	NSP4	0.4671	0.47414	0.59384	0.63595	0.43836	0.61649	0.2752	0.23842	0.47317	0.21903	0.65976	0.68001	0.42271	0.71209	0.31941	0.12373	0.6927	0.31707	0.6168	0.68543	0.34521	0.21028	0.28684	0.09503	0.668
11083	G>T	L37F	NSP6	0.22083	0.27702	0.15833	0.13438	0.37104	0.15852	0.33259	0.41641	0.22154	0.15408	0.17958	0.11959	0.15992	0.25173	0.37323	0.28034	0.0649	0.24387	0.04241	0.0762	0.31922	0.39125	0.6759	0.27119	0.1942
11201	A>G	T77A	NSP6	0.64213	0.51518	0.50802	0.47779	0.32129	0.62527	0.56339	0.172	0.61186	0.09431	0.47461	0.69044	0.2491	0.74151	0.24493	0.05943	0.43245	0.28924	0.60469	0.59731	0.27417	0.42814	0.05257	0	0.4781
11291	G>-	G107-	NSP6	0.09809	0.06291	0.25649	0.195	0.49309	0.09626	0.15535	0.02928	0.37261	0.69623	0.14409	0.10595	0.08477	0	0.37521	0.30617	0.17399	0.65706	0.45814	0.31371	0.34339	0.36114	0.24002	0.68145	0.05466
11292	G>-	G107-	NSP6	0.06606	0.0192	0.16466	0.17073	0.35548	0	0.10945	0	0.08397	0.66789	0.07222	0.07665	0.0672	0	0.20227	0.20945	0.17399	0.56562	0	0.15664	0.21209	0.34254	0.24002	0.35763	0.05466
11295	T>-	F108-	NSP6	0.05878	0.01683	0.13911	0.16686	0.33001	0	0.10219	0	0.08397	0.69059	0.06023	0.07665	0.0672	0	0.18703	0.20945	0.15478	0.53796	0	0.15664	0.21209	0.34254	0.24002	0.35763	0
11296	T>G, T>-	F108L/-	NSP6	0.09532	0.10671	0.26535	0.21855	0.51969	0.14625	0.17304	0.04112	0.40985	1.06149	0.14425	0.07665	0.11691	0.02788	0.46673	0.31846	0.19118	0.76392	0.47995	0.37693	0.34937	0.42441	0.24002	0.75797	0.05466
11418	T>C	V149A	NSP6	0.473	0.46931	0.59464	0.63423	0.44206	0.61266	0.26566	0.23842	0.4696	0.20547	0.64382	0.67878	0.40798	0.75432	0.31941	0.12373	0.6927	0.33035	0.6168	0.68543	0.34521	0.21028	0.26405	0.05423	0.668
14408	C>T	P323L	RdRp	0.09643	0.1688	0.22803	0.11019	0.32851	0.08858	0.21456	0.14973	0.16794	0.13804	0.06023	0.04345	0.29672	0.25173	0.30784	0.05943	0.0649	0.21075	0.07488	0.0762	0.3285	0.41141	0.69298	0.19281	0.2762
15451	G>A	G671S	RdRp	0.58836	0.64525	0.68459	0.66168	0.47603	0.68169	0.60321	0.31178	0.68935	0.23218	0.706	0.41106	0.45721	0.59936	0.46062	0.12373	0.64576	0.35574	0.6866	0.57594	0.36128	0.21028	0.28684	0.05423	0.73936
16466	C>T	P77L	Helicase	0.57781	0.64499	0.68493	0.64981	0.47244	0.68169	0.6015	0.31178	0.68935	0.23218	0.67447	0.41106	0.42987	0.5682	0.43797	0.12373	0.64576	0.35574	0.6866	0.57594	0.36128	0.21028	0.28684	0.05423	0.69197
18877	C>T	Synonymous	Exon	0.27135	0.45056	0.58221	0.25564	0.71311	0.42455	0.46566	0.61125	0.09342	0.3405	0.3804	0.18082	0.32508	0.31449	0.67876	0.68944	0.48856	0.66944	0.15408	0	0.62109	0.42913	0.28684	0.65095	0.51868
21618	C>G, C>-	T19R/-	Spike	0.61687	0.69649	0.69424	0.66525	0.47092	0.64565	0.62389	0.33208	0.69881	0.25732	0.70489	0.38386	0.46373	1.07939	0.46062	0.14251	0.63371	0.36788	0.62249	0.52561	0.42141	0.21028	0.3085	0.10836	0.73936
21765	T>-	I68-	Spike	0.09214	0.05919	0.22344	0.19681	0.43467	0.06848	0.137	0.06264	0.09342	0.68075	0.10518	0.07665	0.0672	0	0.20227	0.26706	0.17399	0.67275	0	0.31371	0.29304	0.37896	0.26405	0.50306	0.05466
21846	C>T	T95I	Spike	0.49689	0.42113	0.24937	0.49858	0.24485	0.49969	0.30412	0.09845	0.43172	0.08477	0.34113	0.71297	0.20125	0.45869	0.21894	0.05943	0.38776	0.17478	0.29306	0.51617	0.13906	0.35329	0.05257	0	0.50085
21872	T>-	W104-	Spike	0.01551	0.02152	0.01262	0.09831	0.03481	0.04375	0.10945	0	0.05328	0.01676	0.1154	0.09167	0.04819	0.7677	0	0.03346	0.03662	0	0.04241	0.10534	0	0.09022	0	0	0.09576
21895	T>C	Synonymous	Spike	0.61142	0.1962	0.1984	0.46263	0.30029	0.24778	0.19274	0.04112	0.14991	0.07054	0.43943	0.11959	0.23034	0.48394	0.03216	0.05943	0.09001	0.24387	0.21902	0.38778	0.25445	0.51534	0	0	0.31864
21987	G>A, G>-	G142D/-	Spike	0.79485	0.3953	0.70642	0.81006	0.44024	0.24609	0.42508	0.15397	0.62208	0.10751	0.37342	0.87608	0.20002	0.87393	0.2336	0.12373	0.79807	0.31707	0.69501	0.8842	0.31113	0.3929	0.05257	0	0.9478
22029	A>-	E156-	Spike	0.61406	0.65597	0.71048	0.6554	0.41524	0.70261	0.1825	0.285	0.18976	0.23218	0.54842	0.43649	0.34302	0.31174	0.42048	0.17579	0.63371	0.36788	0	0.54356	0.37677	0.21028	0.26405	0.05423	0.67946
22030	G>-	E156-	Spike	0.59664	0.6401	0.69686	0.65091	0.41524	0.70822	0.16867	0.285	0.1991	0.23218	0.54842	0.43649	0.33414	0.27366	0.42048	0.14251	0.63371	0.36788	0.04241	0.5848	0.37677	0.21028	0.26405	0.05423	0.67946
22031	T>-	F157-	Spike	0.60235	0.67346	0.68437	0.65091	0.41524	0.69313	0.16867	0.285	0.18976	0.23218	0.54842	0.43649	0.35171	0.28423	0.42048	0.14251	0.63371	0.36788	0.04241	0.52561	0.37677	0.21028	0.26405	0.05423	0.67946
22032	T>-	F157-	Spike	0.60967	0.68957	0.74262	0.66846	0.47422	0.69273	0.19226	0.29591	0.19678	0.23218	0.56653	0.43649	0.35171	0.32413	0.42048	0.14251	0.63371	0.36788	0.04241	0.52561	0.37677	0.21028	0.26405	0.05423	0.67946
22033	C>-	F157-	Spike	0.60949	0.70621	0.74274	0.67733	0.45005	0.78715	0.38529	0.30655	0.21325	0.25117	0.54842	0.42398	0.36953	0.43271	0.42048	0.14251	0.63371	0.36788	0.04241	0.52561	0.37677	0.21028	0.31591	0.05423	0.68251
22034	A>G, A>-	R158G/-	Spike	0.63685	0.92872	0.76092	0.80702	0.48538	1.04209	0.43465	0.34324	0.21945	0.27413	0.56334	0.53622	0.36953	0.4983	0.42048	0.14251	0.69379	0.42817	0.04241	0.78249	0.42387	0.21028	0.26405	0.05423	0.78077
22444	C>T	Synonymous	Spike	0.29383	0.45142	0.58034	0.23637	0.76965	0.45248	0.44954	0.6518	0.14773	0.45028	0.41653	0.19197	0.34984	0.26284	0.70452	0.74302	0.47982	0.81197	0.19623	0	0.73219	0.41141	0.24002	0.76942	0.4781
22917	T>G	L452R	Spike	0.69779	0.70114	0.69929	0.70438	0.56366	0.59888	0.64897	0.38955	0.65476	0.28109	0.68655	0.3315	0.56881	0.51675	0.44641	0.21062	0.6202	0.48147	0.58467	0.34545	0.49384	0.51023	0.3085	0.05423	0.72884
23012	G>C	E484Q	Spike	0.67477	0.2305	0.27189	0.65794	0.35982	0.22794	0.19515	0.07885	0.19218	0.06458	0.55021	0.11959	0.21061	0.20595	0.03216	0.09283	0.12651	0.35578	0.27952	0.34545	0.25445	0.42814	0	0	0.31864
23403	A>G	D614G	Spike	0.08918	0.16754	0.07423	0.08892	0.2735	0.09594	0.21456	0.1573	0.17535	0.13804	0.07905	0.04345	0.29672	0.25173	0.30784	0.05943	0.0649	0.21075	0.07488	0.0762	0.31113	0.41141	0.69298	0.19281	0.22213
23604	C>A, C>G	P681H/R	Spike	0.81242	0.83122	0.89132	0.9359	0.94518	0.73117	0.8514	0.53191	0.88416	0.75325	0.94912	0.37372	0.73028	0.55293	0.82165	0.5294	0.7316	1.02063	0.76675	0.38844	0.76724	0.8576	0.54199	0.63369	0.76149
24410	G>A, G>-	D950N/-	Spike	0.64856	0.72581	0.64974	0.65163	0.4585	0.69249	0.50982	0.28541	0.85053	0.2221	0.90113	0.40763	0.56082	0.70361	0.48851	0.14251	0.63789	0.30337	0.62249	0.52561	0.37677	0.21028	0.12693	0	0.77309
25469	C>T	S26L	ORF3a	0.7663	0.6986	0.71957	0.69245	0.55832	0.61593	0.64897	0.35619	0.65476	0.28684	0.69194	0.35538	0.51725	0.51675	0.44641	0.1774	0.6202	0.48147	0.58467	0.34545	0.49384	0.51023	0.3085	0.05423	0.68251
25563	G>T	Q57H	ORF3a	0.28765	0.45882	0.60102	0.2528	0.76199	0.44381	0.45056	0.63888	0.12801	0.35048	0.40975	0.18082	0.38671	0.30119	0.71135	0.68944	0.47982	0.66944	0.21903	0	0.66976	0.42913	0.3386	0.65095	0.51868
26060	C>T	T223I	ORF3a	0.08362	0.07347	0.14926	0.21962	0.36202	0.02953	0.05406	0.36267	0	0.07488	0.23472	0.02428	0.28684	0.04974	0.39267	0.62056	0.27347	0.21075	0	0	0.11113	0.15514	0	0.35763	0.09576
26735	C>T	Synonymous	Membrane	0.26795	0.44863	0.58349	0.25564	0.72004	0.43616	0.44746	0.61318	0.08397	0.34552	0.3781	0.18082	0.33414	0.28423	0.67876	0.68735	0.47982	0.66383	0.15408	0	0.62109	0.42913	0.26405	0.65645	0.51868
26767	T>C, T>G	I82T/S	Membrane	0.92484	0.74928	0.79834	0.99595	0.65229	0.73239	0.70691	0.34806	0.75549	0.31041	0.91621	0.38386	0.56936	1.15838	0.46923	0.21612	0.70343	0.49911	0.6806	0.66499	0.48473	0.50778	0.3085	0.05423	0.91189
27638	T>C, T>-	V82A/-	ORF7a	0.70457	0.70183	0.69922	0.69709	0.56983	0.61013	0.6478	0.37214	0.66896	0.28109	0.70498	0.3315	0.53316	0.95253	0.44641	0.1774	0.6202	0.48147	0.58467	0.34545	0.49384	0.51023	0.3085	0.05423	0.68251
27752	C>T, C>-	T120I/-	ORF7a	0.60851	0.68964	0.68525	0.65826	0.47619	0.64112	0.61628	0.33208	0.66687	0.25732	0.67879	0.38386	0.48992	0.98545	0.43797	0.20945	0.63371	0.37969	0.62249	0.52561	0.37677	0.21028	0.3085	0.05423	0.69197
27874	C>T	T40I	ORF7b	0.30259	0.45847	0.43206	0.12207	0.10506	0.58661	0.51994	0.142	0.58845	0.0538	0.12531	0.68531	0.09414	0.76694	0.23118	0	0.40081	0.11358	0.56234	0.46401	0.04569	0	0.05257	0	0.29643
28095	A>T, A>-	K68*/-	ORF8	0.05892	0.05028	0.17582	0.15143	0.31219	0.12437	0.06276	0.08141	0.12325	0.89567	0.22282	0.06068	0.10126	0.04974	0.43011	0.16035	0.0732	0.57319	0.15408	0.10534	0.16495	0.3231	0	0.42932	0
28249	A>T, A>-	D119V/-	ORF8	0.60094	0.65951	0.78731	0.77084	0.48753	0.71674	0.67368	0.33967	0.62247	0.23565	0.64587	0.6624	0.38142	0.47505	0.46062	0.12373	0.85603	0.40566	0.68291	0.84253	0.36128	0.21028	0.2666	0.05423	1.05588
28250	T>-	D119-	ORF8	0.53897	0.58944	0.66136	0.60961	0.42922	0.66965	0.21456	0.26811	0.14482	0.19852	0.48804	0.69044	0.32508	0.31449	0.42048	0.12373	0.68666	0.31707	0	0.66015	0.36128	0.21028	0.15841	0	0.50574
28251	T>-	F120-	ORF8	0.55617	0.60434	0.65483	0.61559	0.44762	0.67739	0.2091	0.29702	0.17458	0.19852	0.48408	0.69044	0.32508	0.31449	0.41143	0.12373	0.72301	0.35326	0	0.66015	0.40599	0.21028	0.21063	0.05423	0.50574
28252	T>-	F120-	ORF8	0.53657	0.58867	0.65127	0.60577	0.41807	0.66883	0.2091	0.26811	0.14482	0.19852	0.50236	0.69044	0.32508	0.31449	0.40216	0.12373	0.72301	0.31707	0	0.66015	0.36128	0.21028	0.15841	0.05423	0.50574
28253	C>A, C>T, C>-	F120L/F/-	ORF8	0.806	1.0594	0.98023	0.88971	0.62579	1.21902	0.98145	0.44447	1.16453	0.33644	0.70958	1.02447	0.50971	0.72887	0.51063	0.22698	1.05282	0.49388	0.97927	0.99245	0.4398	0.28612	0.39584	0.13071	1.0999
28280	G>C	D3H	Nucleocapsid	0.27794	0.20557	0.30014	0.31446	0.52571	0.16917	0.18826	0.03246	0.40088	0.76015	0.29583	0.10087	0.12106	0.1054	0.51293	0.30009	0.15478	0.79577	0.54637	0.41417	0.42924	0.54646	0.24002	0.62397	0.09576
28281	A>T	D3V	Nucleocapsid	0.08343	0.07022	0.21619	0.19284	0.40814	0.08483	0.14356	0.02928	0.37159	0.74341	0.13898	0.07665	0.09414	0.05574	0.48151	0.26706	0.15478	0.60673	0.44671	0.33944	0.29304	0.34254	0.24002	0.62397	0.09576
28461	A>G	D63G	Nucleocapsid	0.56932	0.68436	0.68506	0.65199	0.46968	0.68785	0.6224	0.31178	0.875	0.25732	0.71677	0.38386	0.49753	0.59936	0.42048	0.12373	0.63371	0.36788	0.62249	0.52561	0.37677	0.21028	0.26405	0.05423	0.73936
28688	T>C	Synonymous	Nucleocapsid	0.01383	0.00924	0.0153	0.00513	0.01197	0	0.06276	0	0	0	0	0	0	0	0	0	0	0	0	0	0	0	0.6229	0	0
28854	C>T	S194L	Nucleocapsid	0.27314	0.43084	0.54234	0.26033	0.70723	0.42209	0.42503	0.61881	0.09342	0.37669	0.4136	0.18082	0.26638	0.31449	0.67695	0.7196	0.47982	0.66079	0.15408	0	0.61478	0.4624	0.26405	0.63871	0.53077
28881	G>A, G>T	R203K/M	Nucleocapsid	1.02173	1.05196	0.98391	1.03445	1.08218	0.85363	1.23964	0.99106	1.09273	0.77179	1.07226	0.40285	1.15561	0.59562	0.99908	0.8515	0.82105	1.05025	0.74111	0.38844	1.03137	0.98373	0.80484	0.7328	0.96252
28882	G>A	Synonymous	Nucleocapsid	0.6422	0.60181	0.37309	0.51279	0.61666	0.40395	0.81953	0.69671	0.59295	0.64937	0.51885	0.22691	0.78669	0.12046	0.62134	0.69576	0.34186	0.65425	0.5004	0.31371	0.62109	0.52208	0.54106	0.68648	0.43186
28883	G>C	G204R	Nucleocapsid	0.63539	0.59622	0.37678	0.51279	0.63286	0.40368	0.80388	0.68481	0.59295	0.64575	0.54818	0.20282	0.78669	0.12046	0.62134	0.69576	0.34186	0.65425	0.5004	0.31371	0.62109	0.52208	0.59118	0.68648	0.43186
28916	G>T	G215C	Nucleocapsid	0.30781	0.4512	0.43797	0.11019	0.10094	0.59098	0.53337	0.1341	0.58616	0.0538	0.1154	0.70747	0.09414	0.74627	0.23118	0	0.40081	0.11358	0.56234	0.46401	0.04569	0	0.05257	0	0.29643
29402	G>T	D377Y	Nucleocapsid	0.70178	0.71226	0.69659	0.7006	0.59816	0.6151	0.6736	0.38341	0.67288	0.29765	0.70441	0.3315	0.58678	0.51675	0.49336	0.31794	0.6202	0.49873	0.5916	0.34545	0.5152	0.51023	0.3085	0.05423	0.68251

Table S3: Entropy change in Indian SARS-CoV-2 Genomes based on Temporal analysis

Month	Number of Sequences	Change of Entropy Month wise
January-March-2020	191	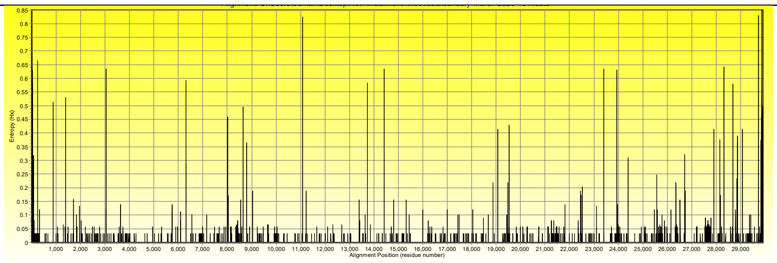
April-2020	441	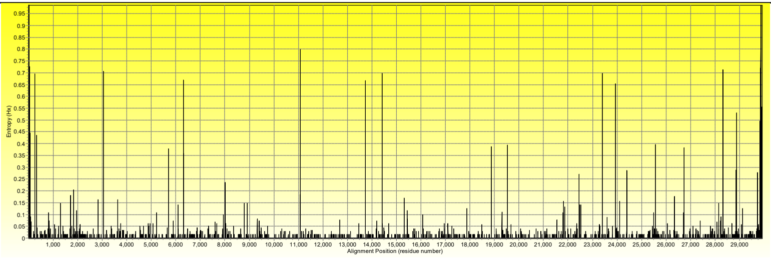
May-2020	977	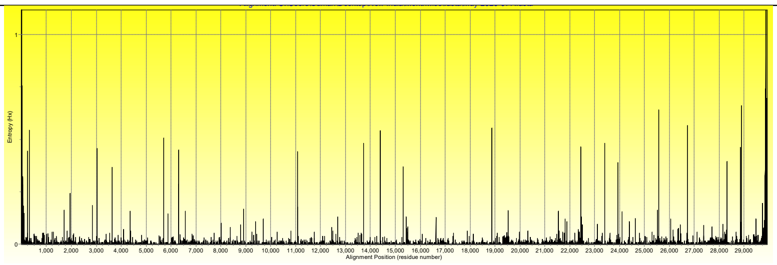
June-2020	1062	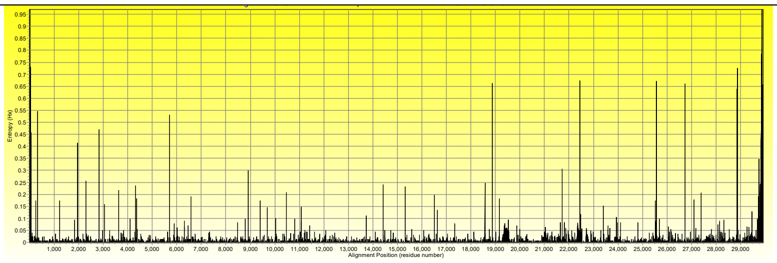
July-2020	683	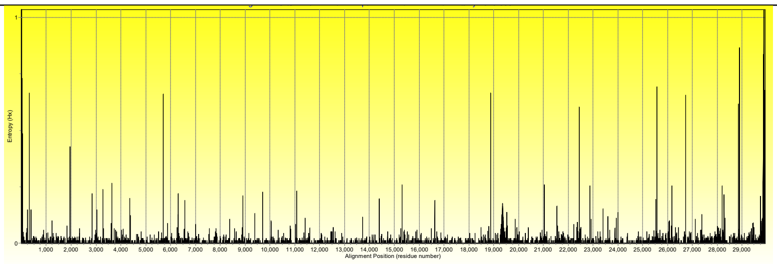
August-2020	632	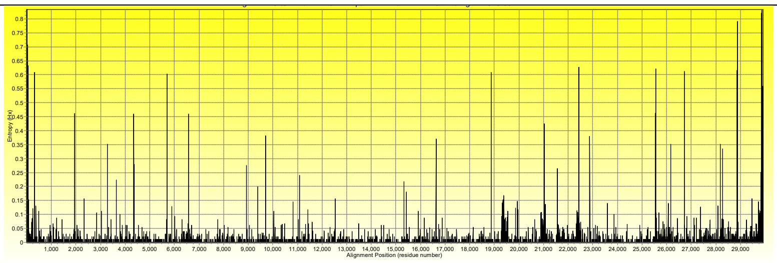

Month	Number of Sequences	Change of Entropy Month wise
September-2020	629	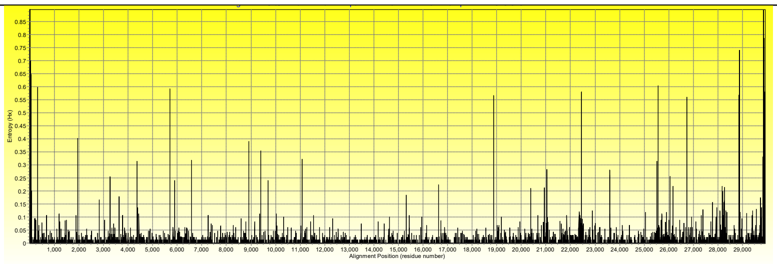
October-2020	380	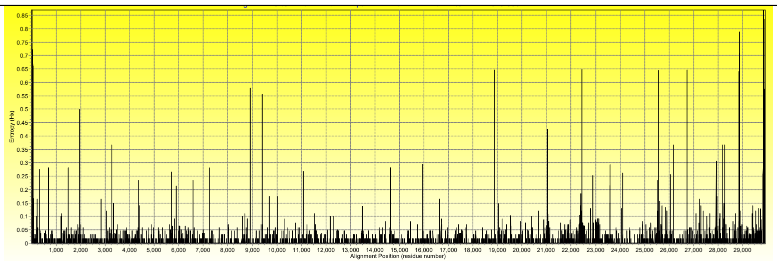
November-2020	452	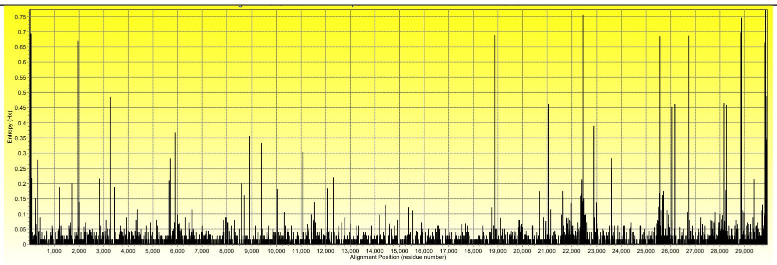
December-2020	983	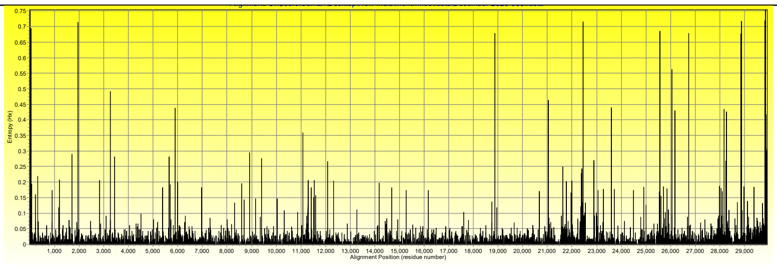
January-2021	500	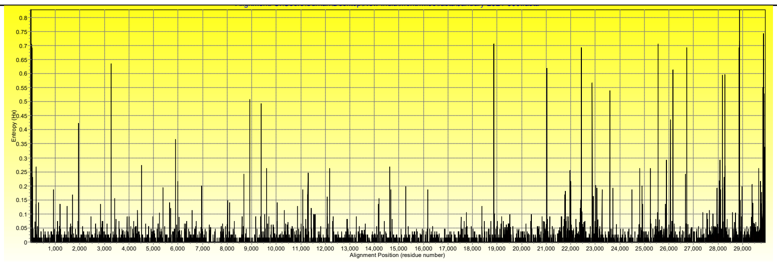
February-2021	980	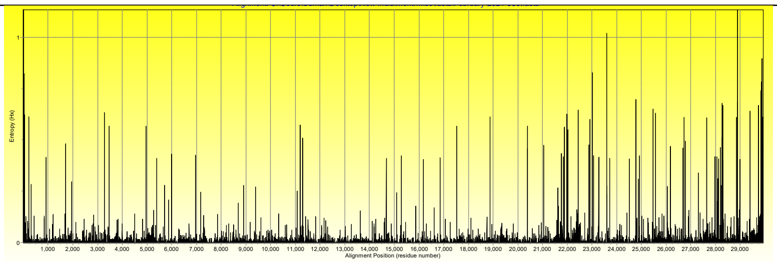

Month	Number of Sequences	Change of Entropy Month wise
March-2021	1907	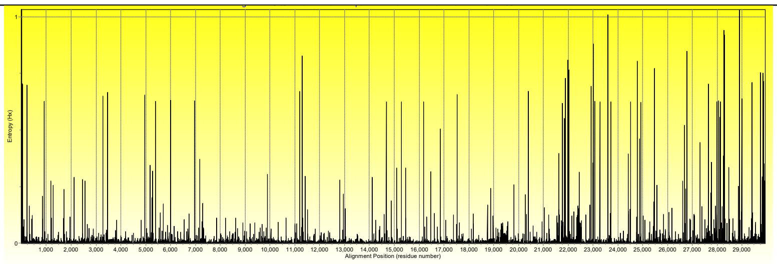
April-2021	3054	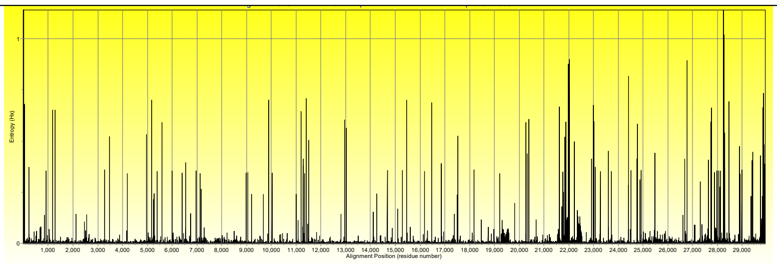
May-2021	2408	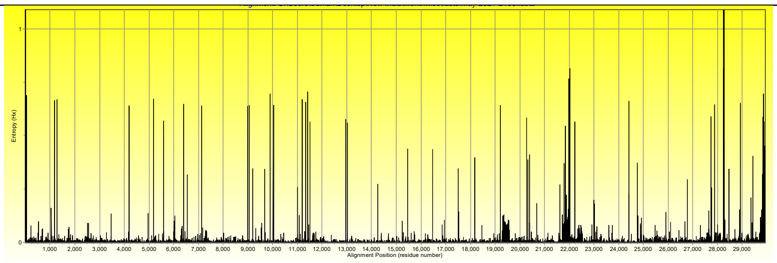
June-2021	1293	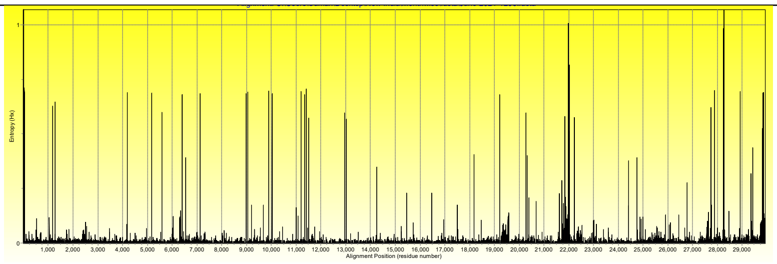
July-2021	632	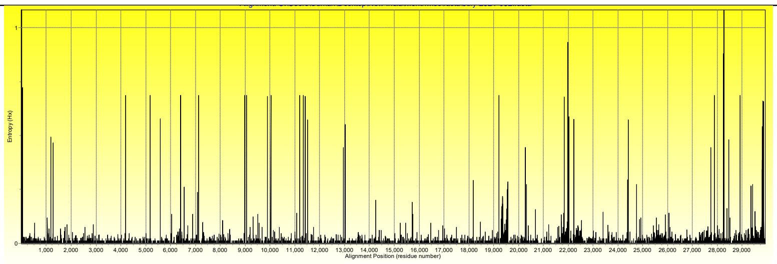
August-2021	15	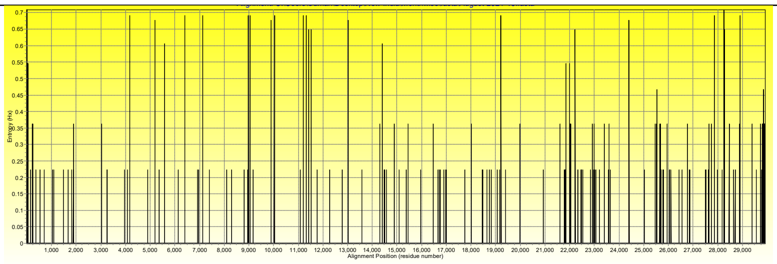

Month	Number of Sequences	Change of Entropy Month wise
September-2021	52	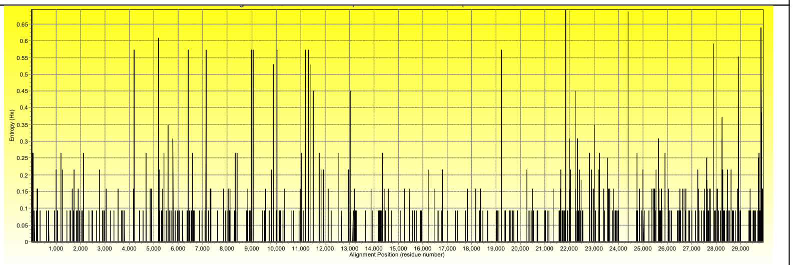

Table S4: Entropy change in Indian SARS-CoV-2 Genomes based on Spatial analysis

State	Number of Sequences	Change of Entropy Month wise
Maharashtra	3674	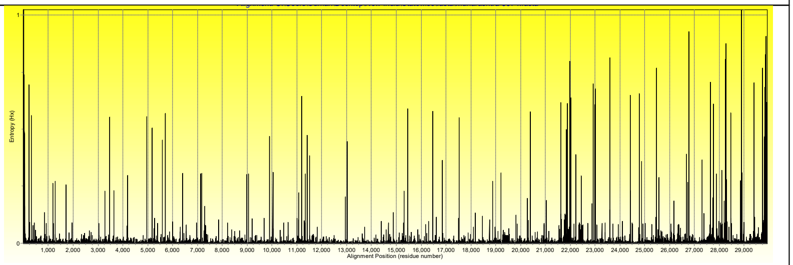
Telangana	2506	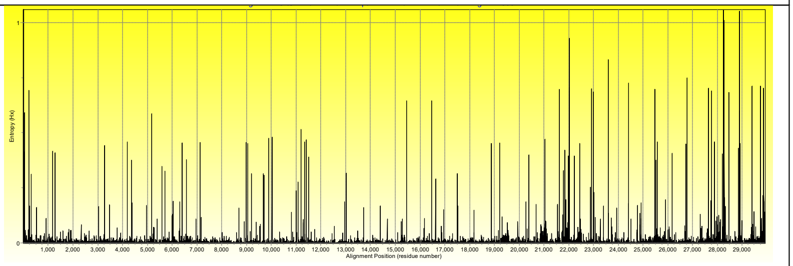
Gujarat	2333	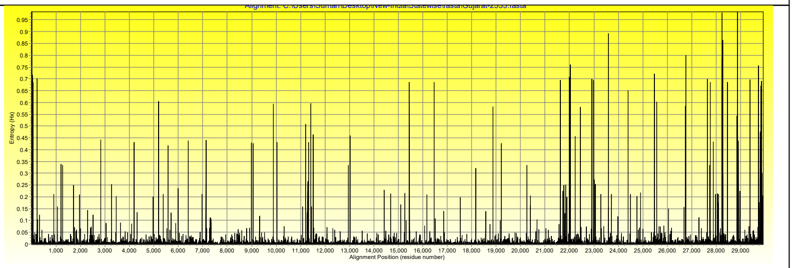
West Bengal	1637	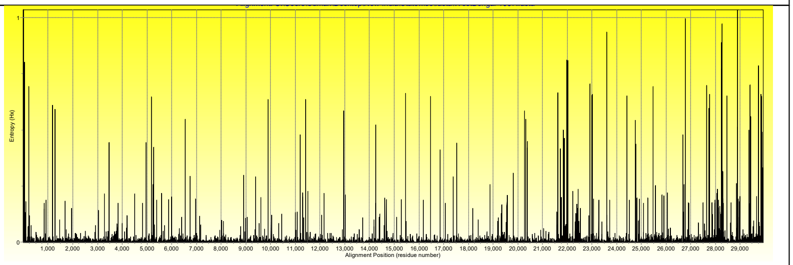
Delhi	1240	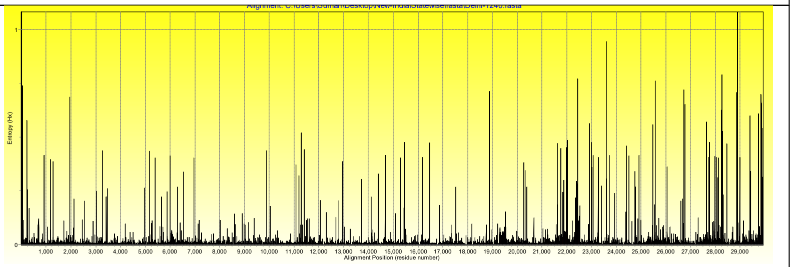
Andhra Pradesh	1077	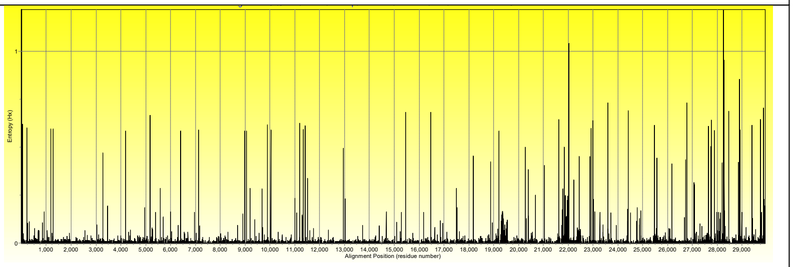

<table>
<tr><th>State</th><th>Number of Sequences</th><th>Change of Entropy Month wise</th></tr>
<tr><td>Karnataka</td><td>520</td><td>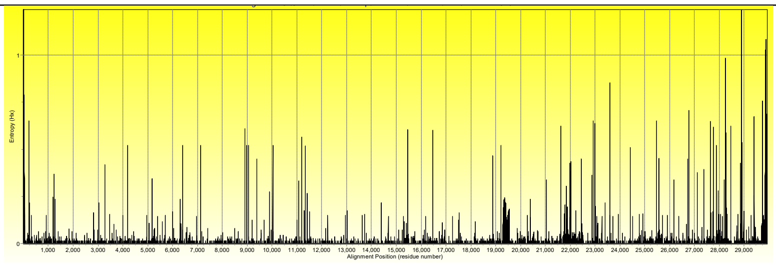
</td></tr>
<tr><td>Rajasthan</td><td>434</td><td>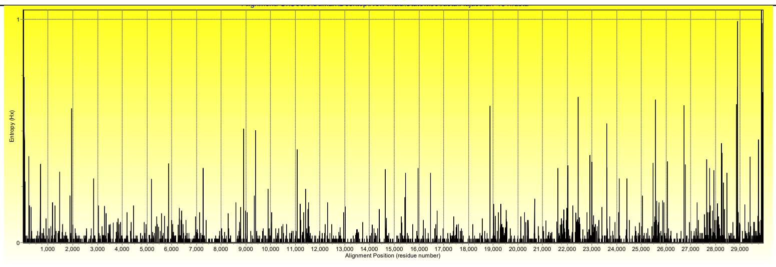
</td></tr>
<tr><td>TamilNadu</td><td>423</td><td>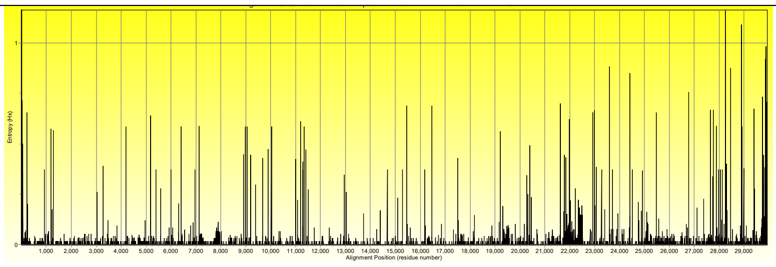
</td></tr>
<tr><td>Punjab</td><td>418</td><td>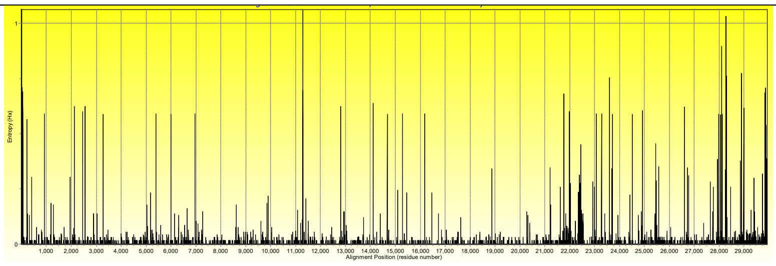
</td></tr>
<tr><td>Chhattisgarh</td><td>364</td><td>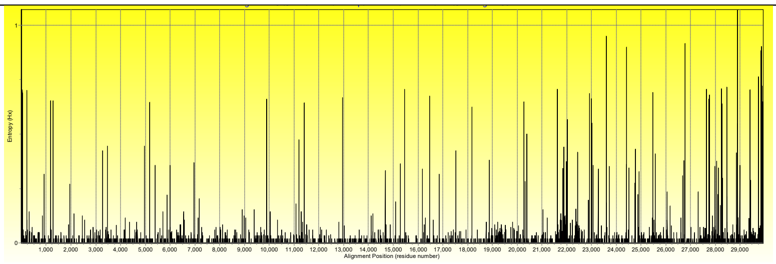
</td></tr>
<tr><td>Manipur</td><td>270</td><td>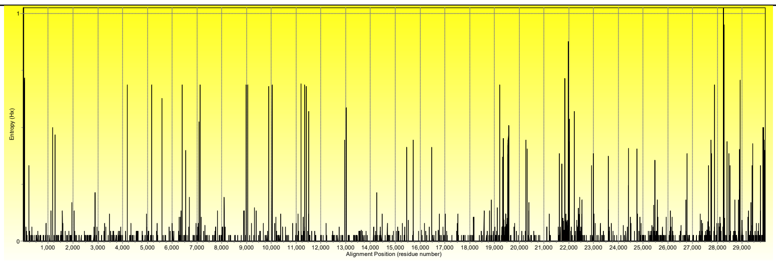
</td></tr>
</table>

State	Number of Sequences	Change of Entropy Month wise
Odisha	238	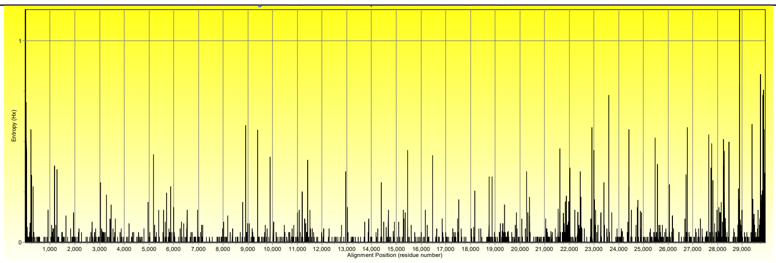
Uttar Pradesh	229	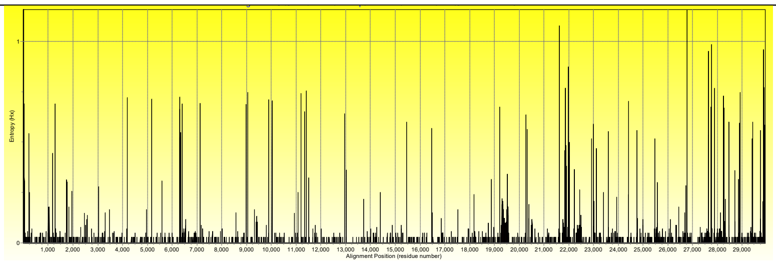
Haryana	193	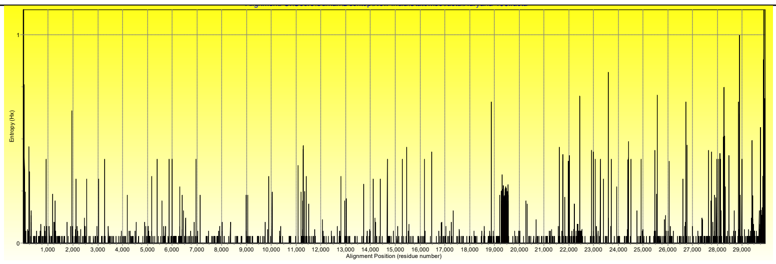
Himachal Pradesh	184	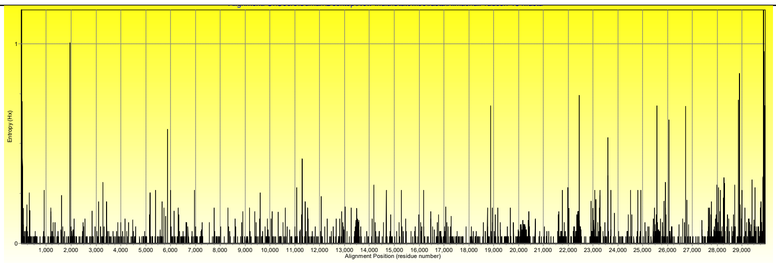
Sikkim	165	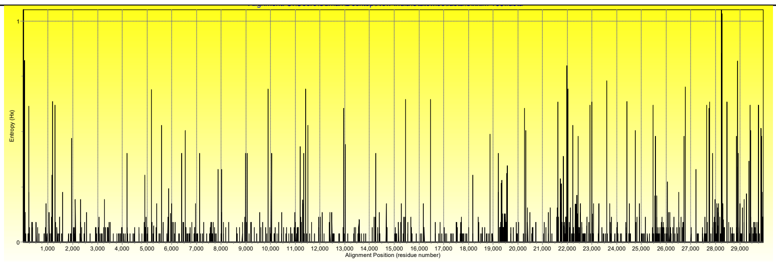
Jammu and Kashmir	164	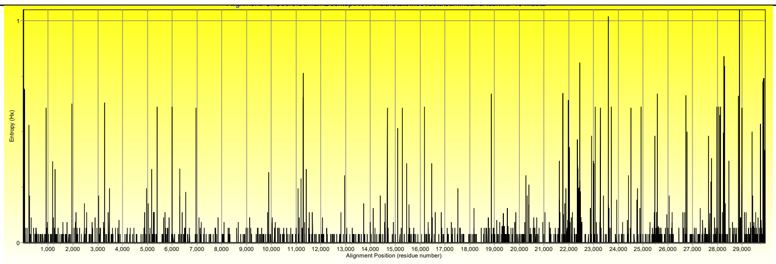

State	Number of Sequences	Change of Entropy Month wise
Puducherry	138	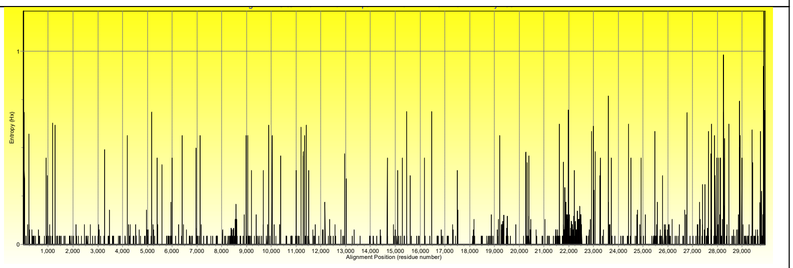
Meghalaya	135	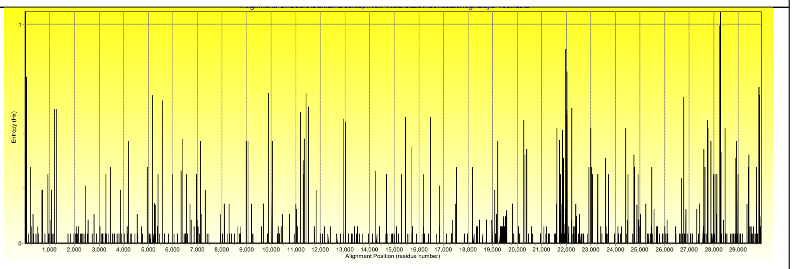
Uttarakhand	126	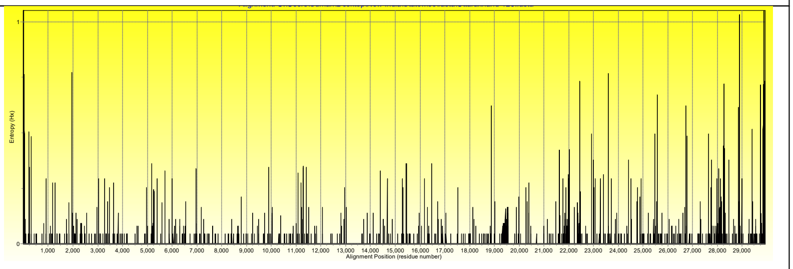
Madya Pradesh	109	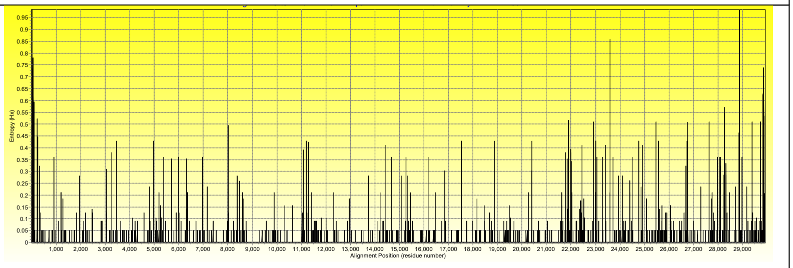
Kerala	106	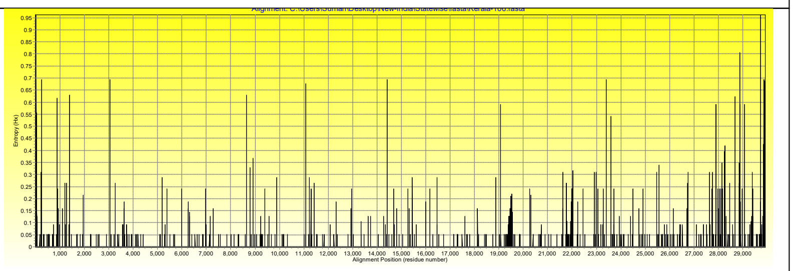
Chandigarh	102	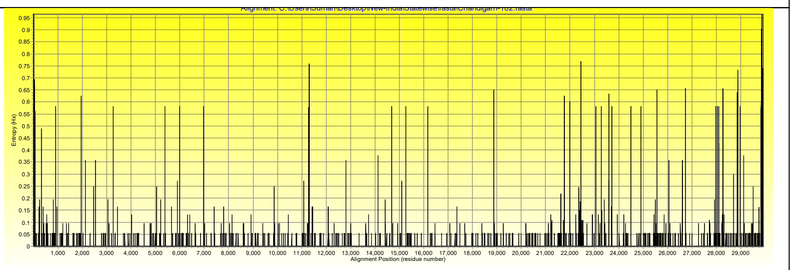

State	Number of Sequences	Change of Entropy Month wise
Assam	101	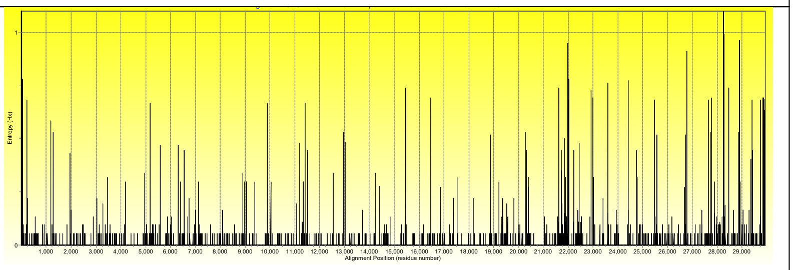
